# Differential regulations of abscisic acid-induced desiccation tolerance and vegetative dormancy by group B3 Raf kinases in liverworts

**DOI:** 10.3389/fpls.2022.952820

**Published:** 2022-07-28

**Authors:** Akida Jahan, Yuto Yamazaki, Mousona Islam, Totan Kumar Ghosh, Nami Yoshimura, Hirotaka Kato, Kimitsune Ishizaki, Akihisa Shinozawa, Yoichi Sakata, Daisuke Takezawa

**Affiliations:** ^1^Graduate School of Science and Engineering, Saitama University, Saitama, Japan; ^2^Biological Research Division, Bangladesh Council of Scientific and Industrial Research (BCSIR), Dhaka, Bangladesh; ^3^Department of Crop Botany, Bangabandhu Sheikh Mujibur Rahman Agricultural University, Gazipur, Bangladesh; ^4^Graduate School of Science, Kobe University, Kobe, Japan; ^5^Department of Bioscience, Tokyo University of Agriculture, Tokyo, Japan

**Keywords:** abscisic acid, liverworts, vegetative dormancy, genome editing, Raf kinase, RNA-seq, *Marchantia polymorpha*

## Abstract

Phytohormone abscisic acid (ABA) plays a key role in stomata closure, osmostress acclimation, and vegetative and embryonic dormancy. Group B3 Raf protein kinases (B3-Rafs) serve as positive regulators of ABA and osmostress signaling in the moss *Physcomitrium patens* and the angiosperm *Arabidopsis thaliana*. While *P. patens* has a single B3-Raf called ARK, specific members of B3-Rafs among six paralogs regulate ABA and osmostress signaling in *A. thaliana*, indicating functional diversification of B3-Rafs in angiosperms. However, we found that the liverwort *Marchantia polymorpha*, belonging to another class of bryophytes, has three paralogs of B3-Rafs, Mp*ARK1*, Mp*ARK2*, and Mp*ARK3*, with structural variations in the regulatory domains of the polypeptides. By reporter assays of the *P. patens ark* line and analysis of genome-editing lines of *M. polymorpha*, we found that these B3-Rafs are functionally redundant in ABA response, with respect to inhibition of growth, tolerance to desiccation and expression of stress-associated transcripts, the majority of which are under the control of the PYR/PYL/RCAR-like receptor Mp*PYL1*. Interestingly, gemmae in gemma cups were germinating only in mutant lines associated with Mp*ARK1*, indicating that dormancy in the gametophyte is controlled by a specific B3-Raf paralog. These results indicated not only conservation of the role of B3-Rafs in ABA and osmostress response in liverworts but also functional diversification of B3-Rafs, which is likely to have occurred in the early stages of land plant evolution.

## Introduction

The phytohormone abscisic acid (ABA) produced upon sensing osmostress caused by decreases in water potential provokes various biochemical changes for protection of tissues from a forthcoming cellular dehydration that might cause severe damage to plant cells (Finkelstein et al., 2002; Sah et al., 2016). In addition to its role in stomata closure, ABA plays a role in the regulation of developmental processes of seeds, including maturation, storage protein accumulation, desiccation tolerance, and dormancy (Kermode, 2005; Ali et al., 2022). ABA is also responsible for maintenance of dormancy in vegetative tissues such as propagules, axillary buds and underground bulbs of perennials (Pan et al., 2021).

Signal transduction of ABA is initiated by the actions of “core signaling components” with the ABA receptor PYR/PYL/RCAR, group A protein phosphatase 2C (PP2C-A), and subgroup III Snf1-related protein kinase2 (SnRK2) as revealed by studies of Arabidopsis (Cutler et al., 2010). PP2C-A blocks the activity of SnRK2 under unstressed conditions, while under a water stress condition, synthesized ABA binds to PYR/PYL/RCAR to inhibit activity of PP2C-A, which results in activation of SnRK2 (Fujii et al., 2009; Ma et al., 2009; Park et al., 2009; Umezawa et al., 2009). The activated SnRK2 in turn phosphorylates ion channels and transcription factors to provoke ABA-specific cellular responses such as closure of stomata and expression of a number of stress-associated transcripts. SnRK2 is thought to be also important for the ABA-regulated dormancy of immature seeds, because a lack of the genes for SnRK2 results in precocious germination in the Arabidopsis silique (Fujita et al., 2009).

ABA serves as a phytohormone not only in angiosperms but also in bryophytes with a gametophyte-dominant life cycle. ABA induces growth secession and freezing and desiccation tolerance and also the formation of vegetative propagules called brood cells in the moss *Physcomitrium patens* (formerly known as *Physcomitrella patens*; Cuming, 2009). Disruption of PP2C-A in the *ppabi1a/b* double mutant caused constitutive desiccation tolerance with enhanced SnRK2 activity, and disruption of SnRK2 in the *ppsnrk2a/b/c/d* quadruple mutant abolished ABA and osmostress responses, indicating the conservation of core signaling mechanisms in the moss (Komatsu et al., 2013; Shinozawa et al., 2019). ABA also plays a role in stress tolerance in liverworts representing another clade of bryophytes. ABA induces desiccation tolerance in the thallus (gametophyte) of *Riccia fluitans* and *Pallavicinia lyellii* (Pence et al., 2005). In the model liverwort *M. polymorpha*, the gemma, a propagule for asexual reproduction, acquires desiccation tolerance by ABA in the presence of sucrose (Akter et al., 2014). We reported that the *M. polymorpha* Mp*ABI1* gene encoding PP2C-A functions as a negative regulator of ABA signaling (Tougane et al., 2010). Furthermore, the disruptant of the Mp*PYL1* gene encoding a PYR/PYL/RCAR-like ABA receptor exhibited strong ABA insensitivity with reduced desiccation tolerance in gemmae (Jahan et al., 2019). Considering that bryophytes are the evolutionarily earliest diverging group of land plants, conservation of the core signaling components of ABA for stress tolerance suggests that acquisition of these components had been crucial for the colonization of land by the common ancestor of land plants (Bowman et al., 2017; Komatsu et al., 2020).

A recent report also indicates that ABA possibly controls dormancy of gemmae in gemma cups in *M. polymorpha*. Gemmae stay dormant as long as they sit inside the gemma cups formed on thalli but grow rhizoids when the gemmae are placed under growth-favorable conditions outside the gemma cups. ABA treatment of *M. polymorpha* gemmae delays rhizoid initiation in a dose-dependent manner (Eklund et al., 2018). Furthermore, the disruptant of Mp*ABI3A*, an ortholog of *ABSCISIC ACID INSENSITIVE3* of Arabidopsis, bore gemmae growing rhizoids in the gemma cups, indicating that the mechanism of dormancy maintained by ABA might be common in liverworts and seed plants (Eklund et al., 2018).

In addition to core signaling components for ABA discovered in angiosperms, our study using *P. patens* revealed that ARK belonging to the group B3 Raf protein kinases (B3-Rafs) is crucial for activation of SnRK2 (Saruhashi et al., 2015). An *ark* mutant designated AR7 showed little response to ABA, cold and hyperosmotic stress, indicating that ARK plays a role in the integration of environmental abiotic signals. AR7 had little SnRK2 activity upon treatment with ABA, cold and hyperosmosis, and a recombinant protein of the ARK kinase domain phosphorylated conserved serine residues in the activation loop of SnRK2 (Saruhashi et al., 2015; Shinozawa et al., 2019). Disruption of ARK in the *ppabi1a/b* mutant abolished SnRK2 activity and ABA-induced gene expression, indicating that ARK is a crucial positive regulator of SnRK2 in ABA and abiotic stress signaling (Islam et al., 2021). Reporter assays of *P. patens* indicated that genes of three of the six B3-Rafs of Arabidopsis designated AtARK1, AtARK2, and AtARK3 can complement the *ark* phenotype (Saruhashi et al., 2015). Disruption of these B3-Rafs in *A. thaliana* resulted in reductions in SnRK2 activity and osmostress response (Katsuta et al., 2020). Our study and studies done by other researchers indicated that the function of B3-Raf as an activator of SnRK2 is conserved in angiosperms (Lin et al., 2020; Takahashi et al., 2020). In addition to B3-Rafs, Arabidopsis genome has six genes for group B2 Raf kinases (B2-Rafs), which have the “PAS domain” that facilitates protein–protein interactions in the N-terminal non-kinase region, and it was shown that the PAS domain is important for dimerization of B2-Rafs (Lee et al., 2014; Nguyen et al., 2019). Disruption of B2-Rafs (RAF10 and RAF11) in Arabidopsis caused loss of ABA response in seed germination, indicating that these kinases might provide an additional mechanism of ABA response in seeds (Nguyen et al., 2019).

In contrast to the *P. patens* genome that has a single gene for B3-Raf (ARK) and no B2-Raf genes, we found that the genome of *M. polymorpha* has multiple genes for B3-Raf and B2-Raf as does Arabidopsis. However, the role of these Raf kinases in liverworts has not been determined. In this study, we generated mutants of group B2-Raf and B3-Raf in *M. polymorpha* by genome editing and analyzed growth and stress responses to ABA. Analyses of the mutant lines revealed differential roles of Raf kinase isoforms in desiccation tolerance and vegetative dormancy in the liverwort.

## Materials and methods

### Culture of plant materials

Male gametophyte of *M. polymorpha* accession Takaragaike-1 (TAK-1) was cultured by planting gemmae on a half-strength B5 agar medium with 1% sucrose (Akter et al., 2014). Protonemata of the *P. patens* for reporter assays were cultured on a cellophane-overlaid BCDAT agar medium (Bhyan et al., 2012).

### Generation of CRISPR Cas-9 genome editing line

For constructs for genome editing lines, double-strand oligonucleotides containing the target guide RNA sequence were fused downstream to the U6 promoter (Sugano et al., 2018) in the pMpGE010 vector containing the Cas9 gene and a hygromycin phosphotransferase gene cassette. For making double mutants, similar constructs were made using pMpGE011, with a mutated acetolactate synthase gene for chlorsufuron-resistance was used (Ishizaki et al., 2015). Agrobacterium (*Rhizobium radiobacter*) C58 strain harboring the above constructs was used for infection of cultured gemmalings of *M. polymorpha* as described by Kubota et al. (2013). After selection on a medium containing 10 mg L^−1^ hygromycin or 0.5 μM chlorsulfuron, gemmae of the generated transgenic lines were individually cultured for further experiments.

### Reporter assays

The cDNA encoding Raf kinases was fused downstream to the rice actin promoter and used for transient reporter assays. The constructs were introduced into protonema cells of the ARK-null AR133 mutant of *P. patens*, in which the CGA codon of Arg1043 has been changed to TGA, with the *proEm-GUS* reporter construct (Marcotte et al., 1989) and the *proUbi-LUC* reference construct (Christensen and Quail, 1996) by particle bombardment using the PDS-1000He particle delivery system (Bio-Rad, Hercules, CA). After bombardment, the protonema cells were cultured at 25°C under continuous light for 1 day on a BCDAT medium with or without 10 μM ABA. Proteins extracted from the protonemata were used for GUS and LUC assays to determine GUS/LUC ratio as previously described (Komatsu et al., 2009).

### Determination of gemma dormancy

Gemmae isolated from gemma cups located at the most basal position in thalli were stained by 15 μM propidium iodide and 0.01% Triton X-100 for 15 min and observed under the binocular fluorescent microscope (Leica M205FA, Leica, Wetzlar, Germany) using the DsRED filter set. The number of gemmae with elongated rhizoids were counted. A total of 60–114 gemmae were used for each data point of three biological replicates.

### Tests for desiccation tolerance

Gemmae were cultured for 1 day in a half-strength B5 liquid medium with or without 1 and 10 μM ABA and transferred onto a sheet of cellophane on wet filter paper in a petri dish. The dish was then placed in an airtight container containing silica gel and kept at 23°C for 2 days in the dark for desiccation treatment. The dried gemmae on the cellophane were rehydrated by immersing in the same liquid medium, and then transferred onto a fresh agar medium and cultured for 10 days to determine survival (Jahan et al., 2019).

### Total RNA extraction and quantitative reverse transcription (qRT-PCR) analysis

Gemmalings cultured for 4 days in liquid medium were treated with or without 10 μM ABA for 6 h for RNA extraction. qRT-PCR analysis was carried out using oligonucleotides for Mp*LEAL1* (*Mapoly0112s0030*, *Mp4g09300*), Mp*LEAL3* (*Mapoly0035s0082*, *Mp6g02960*), Mp*LEAL5* (*Mapoly0087s0015*, *Mp4g05760*), Mp*LEAL6* (*Mapoly0027s0114*, *Mp5g05120*), and Mp*ABI*3 (*Mapoly0086s0035*, *Mp5g08310*; Supplementary Table S1). Quantitative RT-PCR was performed using THUNDERBIRD SYBR qPCR mix (Toyobo, Osaka, Japan). The ABA-unaffected Mp*EF1* (*Mapoly0024s0116*, *Mp3g23400*; Ishizaki et al., 2008) was used for normalization.

### RNA-seq analysis

Quality of total RNA extracted from WT and mutants was confirmed by Bioanalyzer 2100 (Agilent Technologies, Santa Clara, CA, United States). RNA libraries were prepared using 1 μg of total RNA according to the protocol of the NEBNext Ultra II RNA Library Prep Kit for Illumina (New England Biolabs, Ipswich, MA, United States). The library size was 200–700 bp as measures by Bioanalyzer 2100, and the average length of the library was 350 bp. The library concentration was measured by Kapa Library Quantification Kit (Kapa Biosystems, Wilmington, MA, United States) and adjusted to 10 nM. Sequencing of the library was performed on the Hiseq 2500 system (Illumina, San Diego, CA, United States). Conversion of single reads to FASTQ format was performed by bcl2fastq tool version 2.18.0.12 (Illumina). Row read data for each library were stored in the DDBJ (accession number DRA014012). Trimming of the reads, mapping to the *M. polymorpha* genome v3.1 (Phytozome 12)^1^ and extraction of differentially expressed genes (DEGs) were performed using CLC genomics workbench version 12 (Qiagen). In trimming, the following parameters were changed (quality limit = 0.001, number of 5′ terminal nucleotides = 14, number of 3′ terminal nucleotides = 3, and minimum number of nucleotides in reads = 36). Genes with a false discovery rate (FDR) of less than 0.05 and a fold change greater than 2 were defined as DEGs.

## Results

### Characterization of group B2/B3 Raf genes and transient assays in *Physcomitrium patens*

**Figure 1:**
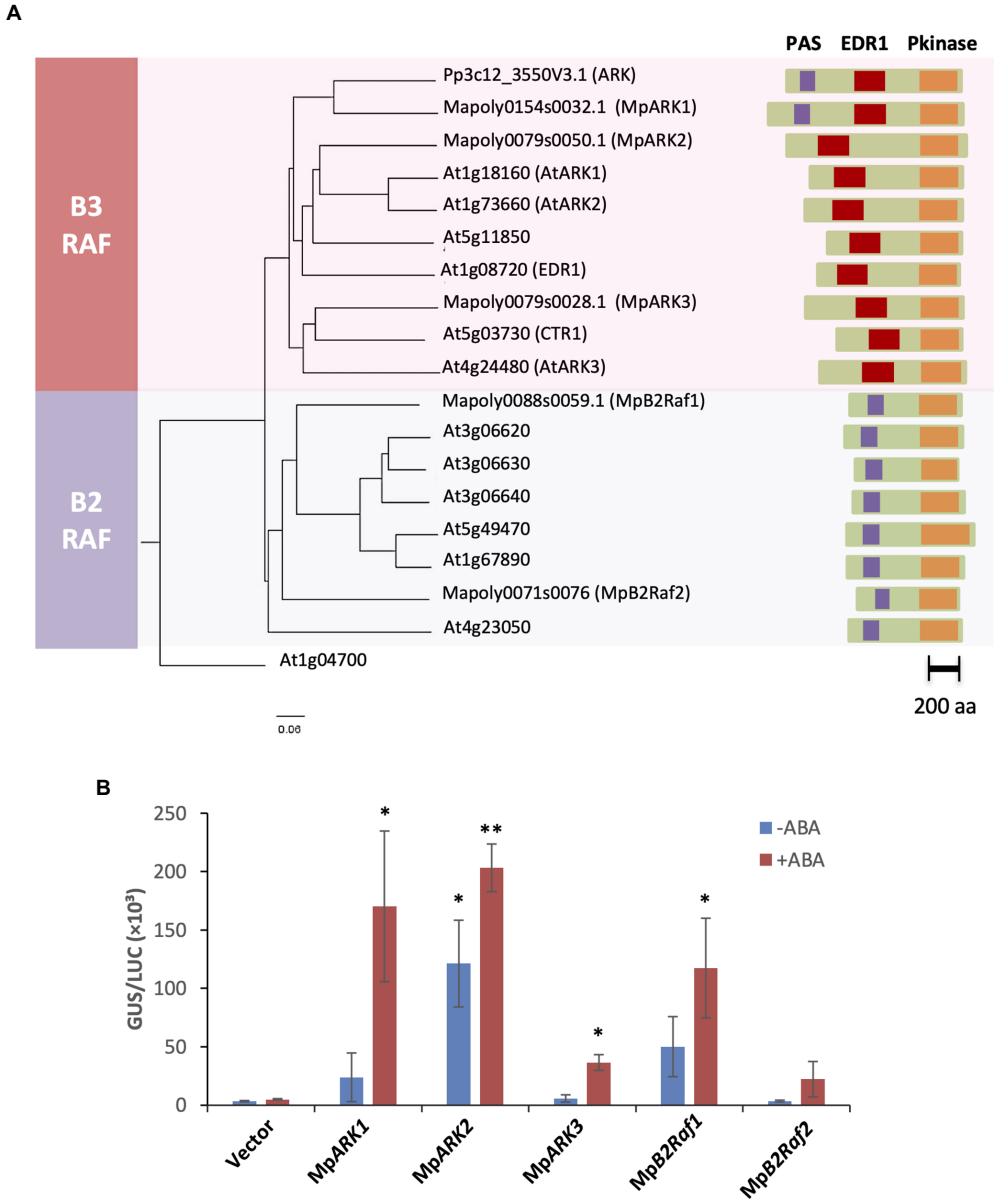
**(A)** Phylogenetic relationships and polypeptide structures of group B2 and B3 Raf kinases. The phylogenetic analysis was conducted by the neighbor-joining method using CLASTALW. Numbers on the branches indicate bootstrap values (100 replicates) and the bar represents the number of amino acid changes per branch length. **(B)** Reporter assays of *ark* mutant of *P. patens* using cDNA constructs of group B2 and B3 Raf kinases of *M. polymorpha*. The constructs of cDNA fused to the rice actin promoter, *proEm-GUS* and *proUbi-LUC* were introduced into protonema cells by particle bombardment. The vector without cDNA was used as a control. The bombarded cells were cultured for 1 day with or without 10-μM ABA before GUS and LUC assays. Levels of gene expression are represented by GUS/LUC ratio. Error bars indicate standard error of the mean. *p < 0.05 and **p < 0.01 in the *t*-test (*n* = 3) compared with WT of the same treatment.

By a BLAST search of the *M. polymorpha* genome database, we identified one gene (*Mapoly0154s0032*, *Mp8g16320*) encoding 1,294 amino acids with the highest similarity to *P. patens* ARK. The gene was designated *M. polymorpha ARK1* (Mp*ARK1*). A search of the Pfam database revealed that, as in the case of *P. patens* ARK, the MpARK1 polypeptide has a PAS domain (amino acids 178–290) toward the N-terminus, a protein kinase domain (amino acids 1019–1266) near the C-terminus and a conserved EDR1 domain (amino acids 585–788) between these domains (Figure 1A). In addition, two other genes for B3-Rafs related to Mp*ARK1* were found in the *M. polymorpha* genome, and these genes were designated as Mp*ARK2* (*Mapoly0079s0050*, *Mp8g15630*) and Mp*ARK3* (*Mapoly0079s0028*, *Mp8g15840*). The polypeptides encoded by these genes had both the EDR1 and C-terminal kinase domains but lacked obvious PAS domains.

To determine whether Mp*ARK1*, Mp*ARK2*, and Mp*ARK3* function in ABA responses, reporter assays were carried out using a *P. patens* mutant lacking ARK. cDNA fused to the rice actin promoter was introduced into protonema cells of the *ARK*-null AR133 mutant of *P. patens*, with constructs of the *β-*glucuronidase gene fused with the ABA-inducible wheat *Em* promoter as a reporter (*proEm-GUS*) and the firefly luciferase gene fused with the rice ubiquitin promoter (*proUbi-LUC*) as an ABA-nonresponsive reference. The assays revealed that the level of ABA-induced *GUS* expression was very low in AR133 but that the expression was restored by the introduction of Mp*ARK1* and Mp*ARK2* and to a lesser extent, by the introduction of Mp*ARK3* (Figure 1B).

We also found that the *M. polymorpha* genome has two group B2-Raf genes, which were designated Mp*B2Raf1* (*Mapoly0088s0059*, *Mp7g02280*) and Mp*B2Raf2* (*Mapoly0071s0076*, *Mp5g15330*). Polypeptide structures of these kinases were similar to those of Arabidopsis B2-Rafs, i.e., they had the C-terminus protein kinase domain and the N-terminus PAS domain but lacked the EDR1 domain in between (Figure 1A). Transient expression of these genes in the *P. patens* AR133 mutant indicated that these kinases also restored the ABA-induced gene expression, with a greater effect of Mp*B2Raf1* compared with Mp*B2Raf2* (Figure 1B).

### Analysis of ABA responses in genome-editing lines of group B3-Raf genes

To determine the role of B3-Rafs in *M. polymorpha*, we transformed a male wild-type line (WT) using genome-editing constructs targeting Mp*ARK1*, Mp*ARK2*, and Mp*ARK3* to generate mutant lines. The 18-nucleotide target sequences were designed in the kinase domain of each gene and fused between the Mp*U6* promoter and the gRNA backbone sequence in the MpGE10 vector carrying a hygromycin resistant marker. By Agrobacterium-mediated transformation, we obtained Mp*ark1^ge^*, Mp*ark2^ge^*, and Mp*ark3^ge^* mutant lines (Supplementary Figure S1). When gemmae of these lines were planted on a medium containing ABA, all of the lines showed reduced growth sensitivity to ABA at 1 μM in comparison with WT. However, these single mutant lines retained sensitivity to a higher concentration (10 μM) of ABA (Supplementary Figure S2).

**Figure 2:**
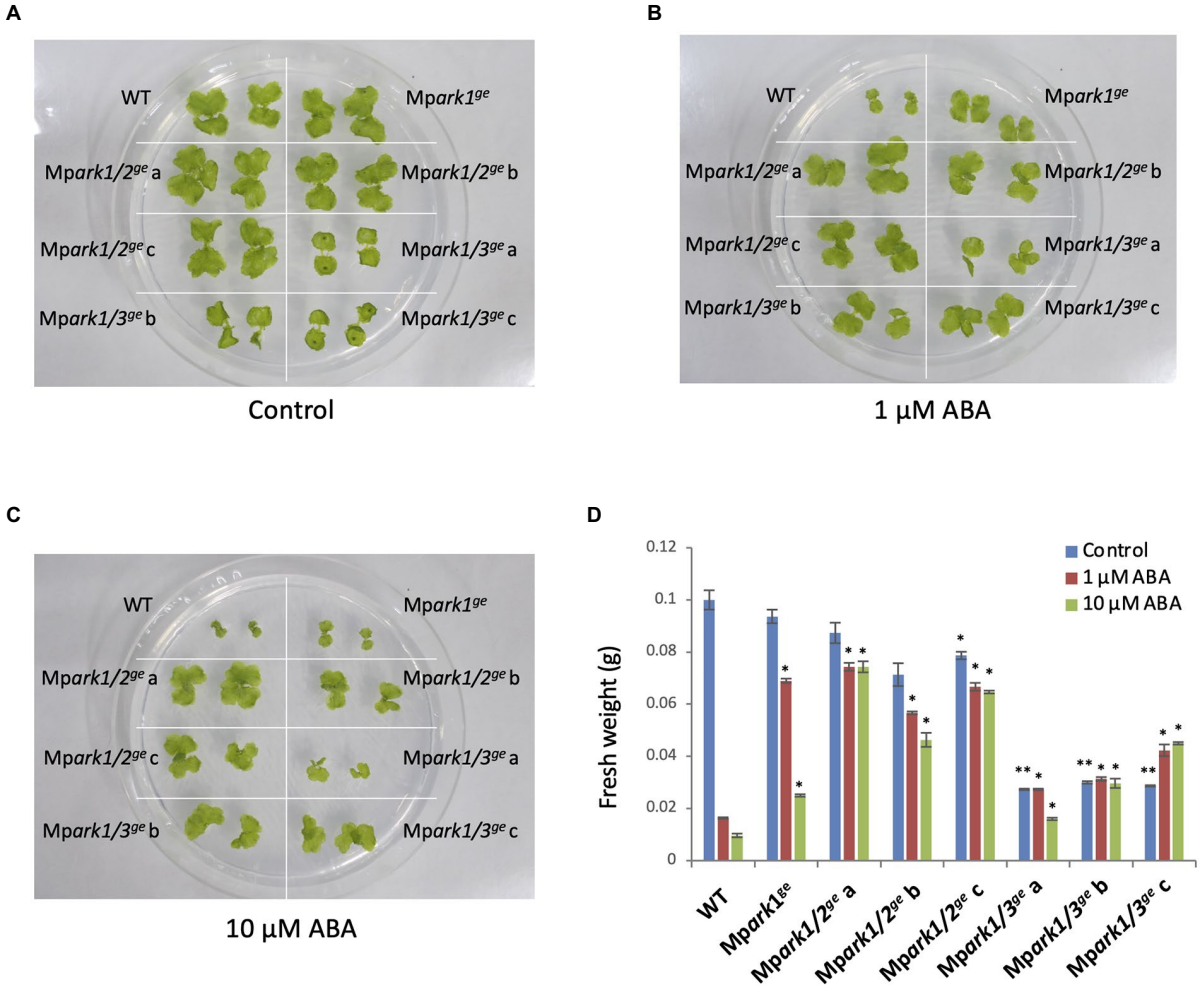
Effect of ABA on growth of gametophytes of *M. polymorpha*. Gemmae of wild type (WT) and three each of double mutant lines of Mp*ARK1* and Mp*ARK2* (Mp*ark1/2^ge^*a to c) and Mp*ARK1* and Mp*ARK3* (Mp*ark1/3^ge^*a to c) were grown on medium without ABA (control) **(A)** or with 1-μM **(B)** or 10-μM **(C)** ABA for 14 days. **(D)** Fresh weight of the gemmalings was plotted in histograms. Error bars indicate the standard error (*n* = 3). *p < 0.05 and **p < 0.01 in the *t*-test compared with WT of the same treatment.

Thus, we made double mutant lines by transforming the Mp*ark1^ge^* line using the MpGE11 vector carrying a chlorsulfuron-resistance marker gene and obtained Mp*ark1/2*^ge^ and Mp*ark1/3*^ge^ lines (Supplementary Figure S1). When gemmae of three independent lines of both mutants were grown on media containing 1 and 10 μM ABA, the plants were resistant to 10 μM ABA (Figure 2), although some growth retardation with or without ABA was observed in the Mp*ark1/3*^ge^ lines. These results indicated that B3-Rafs play a role in ABA responses in gemmalings of *M. polymorpha*. We also obtained both single and double mutants of Mp*B2Raf1* and Mp*B2Raf2* by genome editing (Supplementary Figure S3a). Growth analysis of gemmae of these lines, however, indicated that their ABA response was similar to that of WT (Supplementary Figure S3b).

### Vegetative dormancy and desiccation tolerance in the B3-Raf genome-editing lines

**Figure 3:**
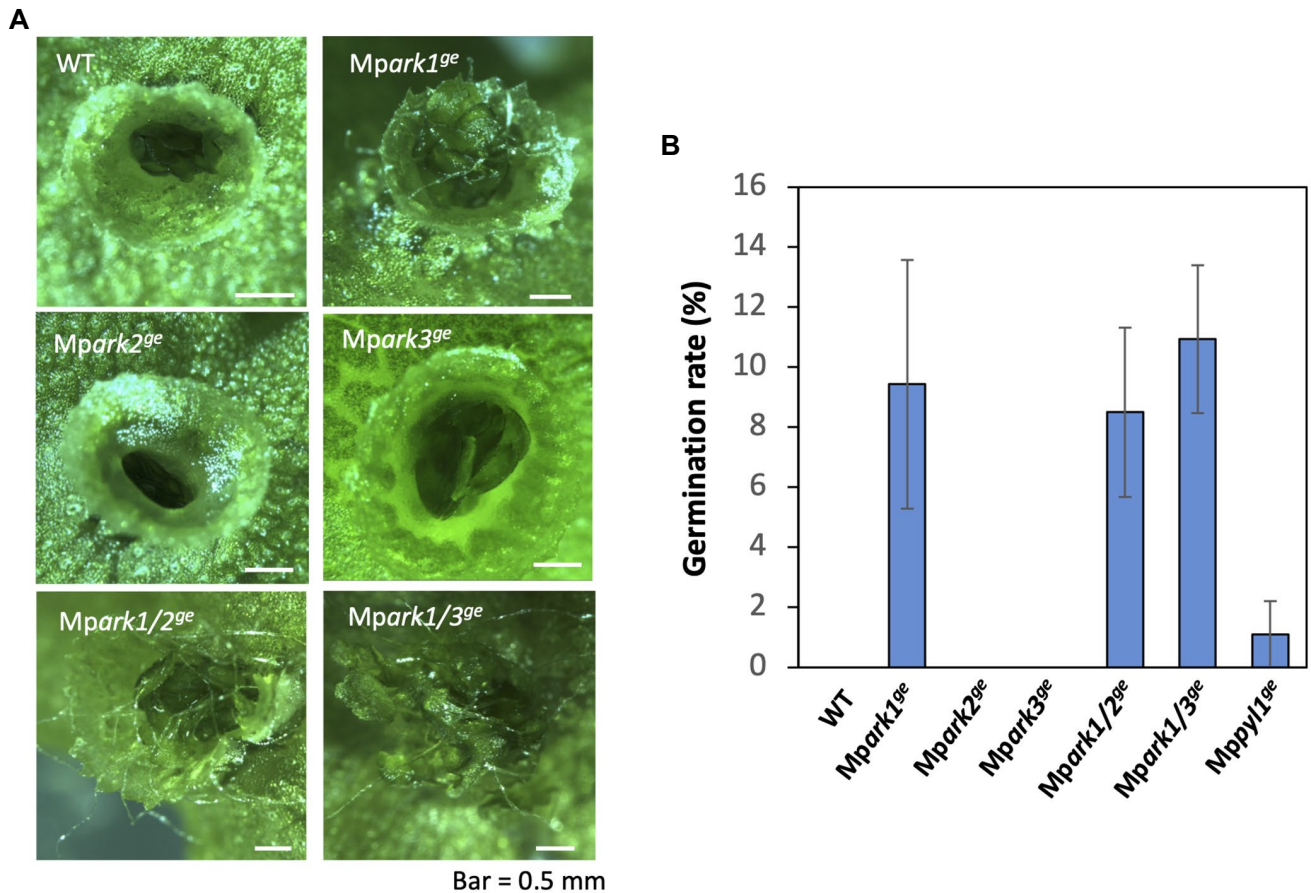
Comparison of dormancy of gemmae in gemma cups. **(A)** Appearance of gemmae in a gemma cup of WT and the B3-Raf genome-editing lines of *M. polymorpha*. Germination of rhizoids was frequently observed in gemmae of Mp*ark1^ge^*, Mp*ark1/2^ge^*, and Mp*ark1/3^ge^* lines while gemmae of WT and Mp*ark2^ge^* and Mp*ark3^ge^* remained dormant within the gemma cup. **(B)** Germination rate of gemmae in gemma cup of WT and genome editing lines. Gemmae isolated from a gemma cup located at the most basal position in the thallus were used for analysis. Germination of rhizoids from gemmae was analyzed by staining with propidium iodide, and the percentages of gemmae growing rhizoids were plotted in histograms. The experiment was carried out on a date different from that shown in Supplementary Figure S4. Error bars indicate +/− SE of the mean (*n* = 3).

Our observations of the generated genome-editing lines indicated that many gemma germinate to form rhizoids in gemma cups in Mp*ark1^ge^*, Mp*ark1/2^ge^*, and Mp*ark1/3^ge^* but not in WT, Mp*ark2^ge^* and Mp*ark3^ge^* (Figure 3A). Counting the number of gemmae growing rhizoids in gemma cups revealed that germination rates of Mp*ark1^ge^*, Mp*ark1/2^g^*,*^e^* and Mp*ark1/3^ge^* were similar (Figure 3B), while there was no germination of gemmae at a similar growth stage in WT, Mp*ark2^ge^* and Mp*ark3^ge^*. We compared the germination rates of these genome-editing lines with the germination rate of Mp*pyl1^ge^* lacking the major PYR/PYL/RCAR in gametophytes (Jahan et al., 2019), which shows strong ABA insensitivity. Interestingly, the germination rate of the gemmae of Mp*pyl1^ge^* was greater than that of WT but much less than that of Mp*ark1^ge^*, Mp*ark1/2^ge^* and Mp*ark1/3^ge^* (Figure 3B; Supplementary Figure S4).

**Figure 4:**
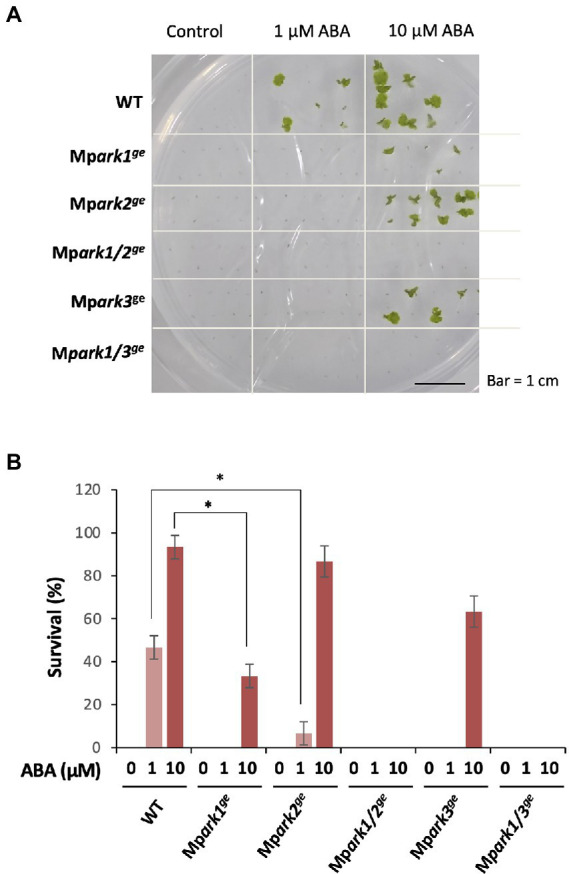
Effect of ABA on desiccation tolerance in *M. polymorpha.* Gemmae of wild type (WT) and the B3-Raf genome-editing lines were cultured for 1 day with or without 1 or 10 μM ABA in liquid medium. The gemmae were transferred to a container containing silica gel and dried for 2 days. After rehydration, the gemmae were transferred onto a fresh agar medium and cultured for 10 days to determine survival. **(A)** Growth appearance of gemmalings after culture for 10 days. **(B)** Survival rate was plotted in histograms. Error bars indicate +/− SE. *p < 0.05 by *t*-test (*n* = 3).

We also examined desiccation tolerance in the gemmae of WT and the mutant lines. Gemmae of these plants were incubated with or without 1 and 10 μM ABA for 1 day and dried for 2 days in a chamber containing dried silica gel. Determination of survival by rehydration and reculture on a fresh medium revealed that all mutant lines showed reduced desiccation tolerance in comparison with that of WT (Figure 4A; Supplementary Figure S5), i.e., survival was enhanced by 1 and 10 μM ABA in WT as we reported previously (Jahan et al., 2019), but it was not enhanced by 1 μM ABA in the single mutants Mp*ark1^ge^*, Mp*ark2^ge^*, and Mp*ark3^ge^* and even by 10 μM ABA in the Mp*ark1/2^ge^* and Mp*ark1/3^ge^* double mutants (Figure 4B).

### RNA-seq analysis of genome-editing lines

**Figure 5:**
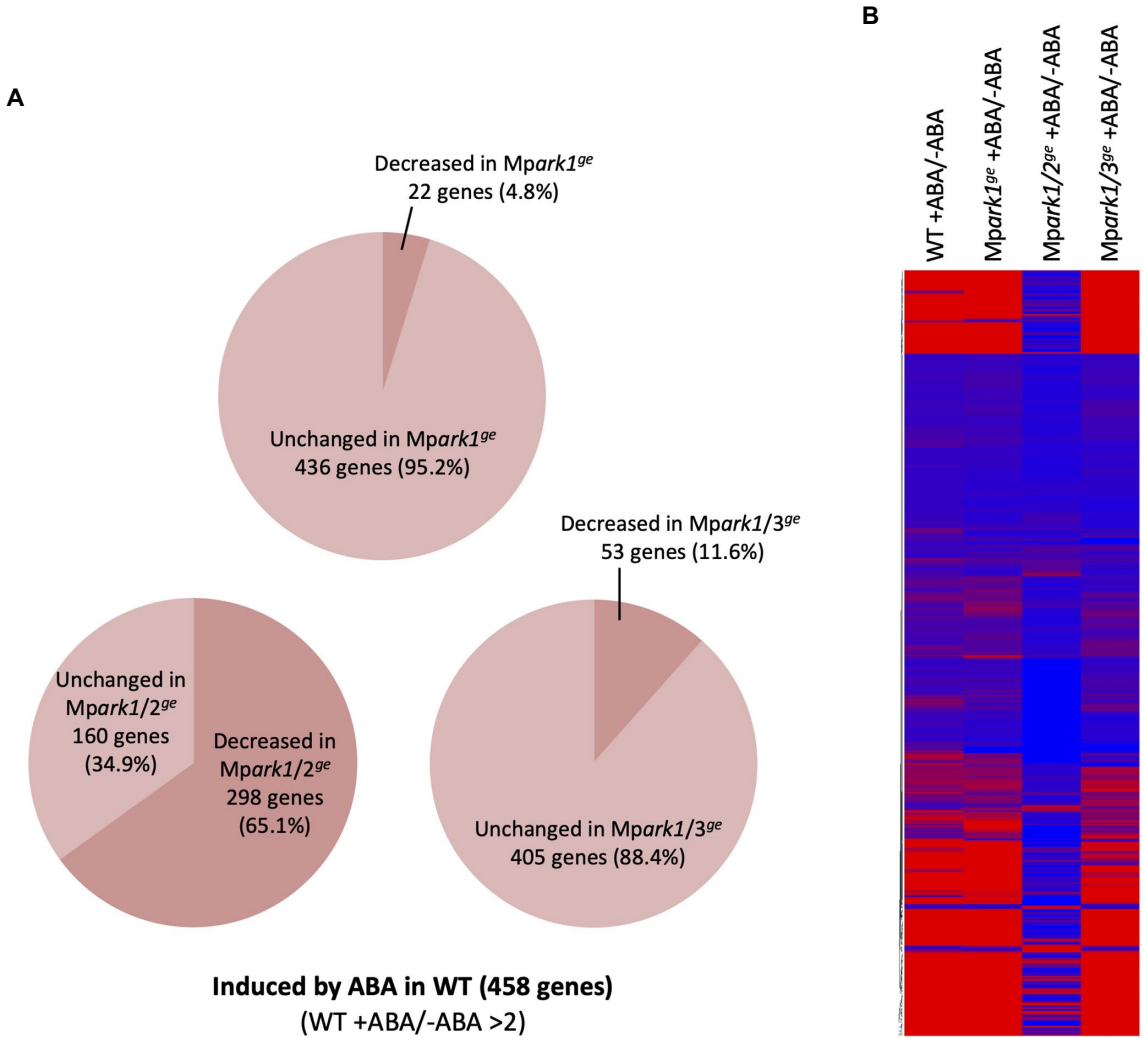
Profiles of ABA-responsive gene expression in wild type (WT) and the B3-Raf genome-editing lines. **(A)** Percentages of ABA-induced genes (>2) for which expression was reduced (<2) in the genome-editing lines. **(B)** Heat map showing ABA-induced and ABA-reduced genes in WT and the genome editing lines. The fold change used for the color key was calculated by comparing values of ABA-treated and control samples.

To reveal global changes in gene expression controlled by B3-Rafs, we analyzed ABA-responsive transcriptomes in representative genome-editing lines. RNA-seq analysis revealed that 458 genes were induced more than two-fold by 6-h treatment with 10 μM ABA in WT. Expression of 22 (4.8%) of the genes was decreased to less than half in ABA-treated Mp*ark1^ge^* compared with that in ABA-treated WT, while expression of 298 (65.1%) and 53 (11.6%) of the genes was decreased in Mp*ark1/2^ge^* and Mp*ark1/3^ge^*, respectively (Figure 5A; Supplementary Figure S6). A heat map of gene expression also indicated that the majority of genes induced by ABA in WT were not induced in Mp*ark1/2^ge^* (Figure 5B). We also analyzed the expression of ABA-repressed genes. Of 87 ABA-repressed genes detected in WT, expression of 24 (27.5%), 57 (65.5%), and 44 (50.6%) of the genes was increased in Mp*ark1^ge^*, Mp*ark1/2^ge^*, and Mp*ark1/3^ge^*, respectively (Supplementary Figure S7).

**Figure 6:**
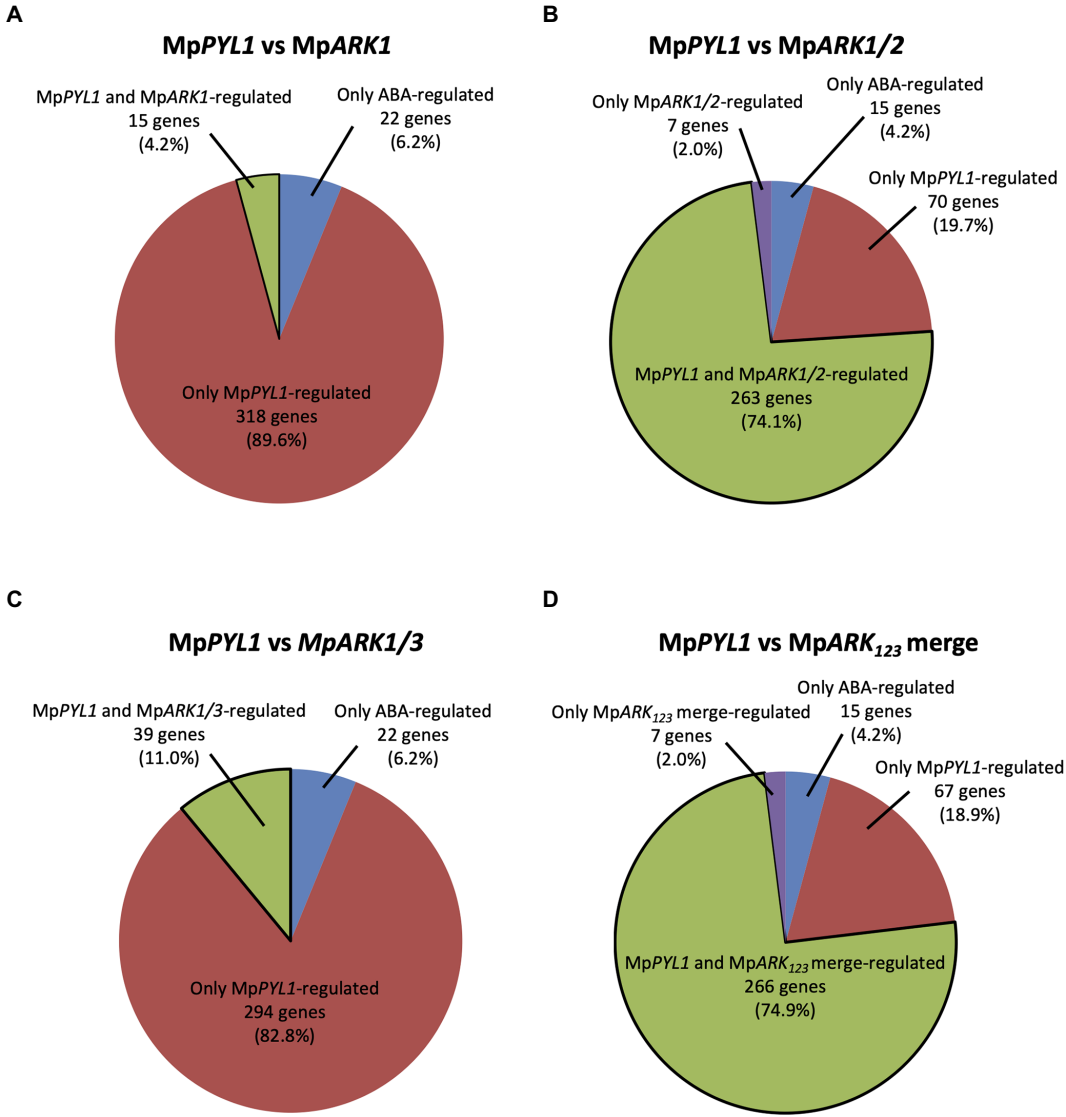
Relationships between ABA-induced genes mediated by the ABA receptor MpPYL1 and B3-Rafs. Using ABA-induced 355 genes commonly identified in this study and our previous study (Jahan et al., 2019), percentages of genes under the control of MpPYL1, B3-Rafs and both are analyzed. Genes for which expression in Mp*pyl1^ge^*, Mp*ark1^ge^*, Mp*ark1/2^ge^*, and Mp*ark1/3^ge^* is less than half of WT are represented as Mp*PYL1*-, Mp*ARK1*-, Mp*ARK1/2*-, and Mp*ARK1/3*-regulated genes **(A–C)**. The result of analysis against the merge of Mp*ark1^ge^*, Mp*ark1/2^ge^*, and Mp*ark1/3^ge^* (Mp*ARK_123_* merge) is also shown **(D)**.

We previously showed that the expression of approximately 80% of ABA-induced genes was decreased in the Mp*pyl1^ge^* mutant (Jahan et al., 2019). Reanalysis of the extracted 333 ABA-induced genes previously identified as genes regulated by Mp*PYL1* indicated that expression of 15 (4.5%), 263 (79.0%), and 39 (11.7%) of the genes was reduced in Mp*ark1^ge^*, Mp*ark1*/2*^ge^*, and Mp*ark1*/3*^ge^,* respectively. This corresponded to 4.2%, 74.1%, and 11.0% of ABA-induced genes commonly identified in this study and the study by Jahan et al. (2019); (Figures 6A–C). A comparison with the merge of the results of Mp*ark1^ge^*, Mp*ark1*/2*^ge^*, and Mp*ark1*/3*^ge^* indicated that the expression of 266 genes (79.9%) regulated by Mp*PYL1* was reduced in either of the B3-Raf mutants (Figure 6D), indicating that the majority of B3-Raf-regulated genes are under the control of Mp*PYL1*.

**Figure 7:**
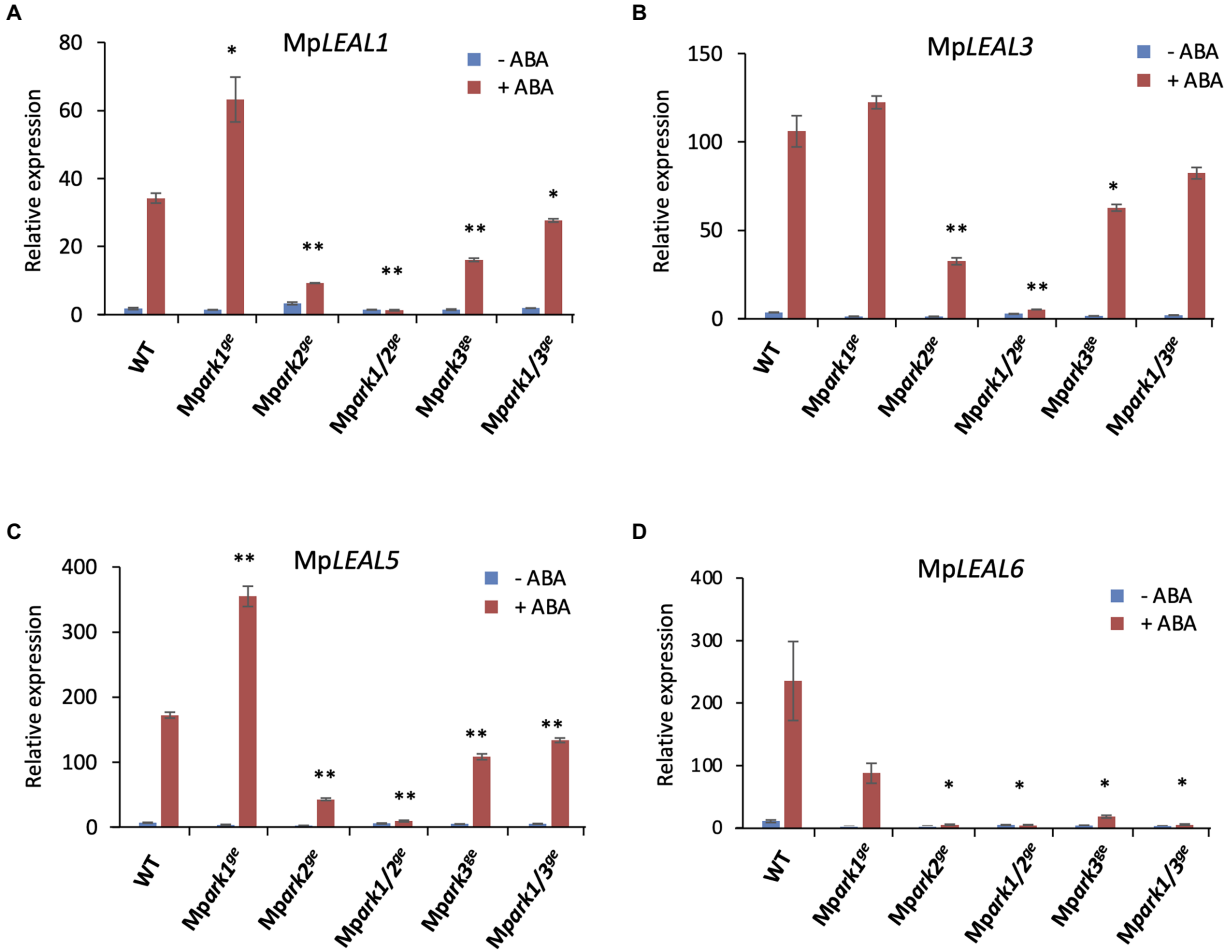
ABA-induced expression of *LEA-*like genes in wild type (WT) and B3-Raf genome editing lines. Quantitative RT-PCR analysis was carried out using RNA isolated from gemmalings treated with or without 10 μM ABA. Transcripts for Mp*LEAL1* (*Mapoly0112s0030*) **(A)**, Mp*LEAL3* (*Mapoly0035s0082*) **(B)**, Mp*LEAL5* (*Mapoly0087s0015*) **(C)**, and Mp*LEAL6* (*Mapoly0027s0114*) **(D)** were analyzed. Relative values were determined by SYBR green-based quantitative PCR analysis using Mp*EF1* (*Mapoly0024s0116*) unaffected by ABA as a reference. Error bars indicate +/− SE. *p < 0.05, **p < 0.01 by *t*-test (*n* = 3) compared with WT of the same treatment.

Previous studies shed light on the role of ABA-induced *Late Embryogenesis Abundant* (*LEA*)-like transcripts in desiccation tolerance in bryophytes (Akter et al., 2014; Hatanaka et al., 2014). *LEA*-like genes encoding hydrophilic proteins are thought to be important for protection from dehydration damage (Shih et al., 2008). We reported that *M. polymorpha LEA-like* (Mp*LEAL*) transcripts are induced by ABA in WT but not in Mp*pyl1^ge^*, which is desiccation-sensitive (Jahan et al., 2019). Quantitative RT-PCR analysis for four representative Mp*LEAL* genes revealed that ABA-induced expression of all four transcripts was very little in Mp*ark1*/*2^ge^* in comparison with that in WT. Reductions in the expression of these Mp*LEAL* genes to various extents were observed in Mp*ark2^ge^*, Mp*ark3^ge^*, and Mp*ark1*/*3^ge^*. In contrast, there was no significant decrease in any of these Mp*LEAL* transcripts in the Mp*ark1^ge^* single mutant (Figure 7).

## Discussion

### Roles of B3 Raf kinases in ABA response are conserved in embryophytes

Sequencing the whole genomes of various organisms has revealed the presence of signal transduction processes for ABA in both vascular and nonvascular plants, indicating that the basic toolkit for ABA signaling has been acquired in the common ancestor of embryophytes (Rensing et al., 2008; Takezawa et al., 2011; Bowman et al., 2017). Identification of the genes for the functional ABA receptors in bryophytes, comprising the earliest divergent groups of land plants, indicates that the acquisition of the receptors might have been the key evolutionary event for the establishment of the mechanism for PP2C-A-mediated SnRK2 regulation in the common ancestor of land plants (Jahan et al., 2019; Sun et al., 2019; Komatsu et al., 2020). Identification of B3-Raf as a positive regulator of SnRK2 has extended our understanding of the integrated regulation of ABA and osmostress signals (Saruhashi et al., 2015; Katsuta et al., 2020; Takahashi et al., 2020). In this study, we demonstrated that the function of B3-Rafs in ABA and abiotic stress response is conserved in liverworts, which are recognized as a distinct lineage of basal land plants, by reporter assays and analysis of mutant lines of *M. polymorpha*. ABA-induced growth retardation, gemma dormancy and desiccation tolerance are important adaptive mechanisms to survive the period of water scarcity. Reversal of phenomena by lack of B3-Rafs indicate that the B3-Rafs are important components for survival in the terrestrial environment. Taken together with the results of our previous studies showing complementation of *P. patens ark* mutant with B3-Raf genes of *A. thaliana* and the lycophyte *Selaginella moellendorffii* (Saruhashi et al., 2015), the results obtained in this study indicate that the role of B3-Rafs in stress signaling is conserved in embryophytes.

The presence of three B3-Rafs in *M. polymorpha* with structural variations provides new insights into the evolutionary diversity of B3-Rafs. Identification of MpARK2 and MpARK3, B3-Rafs phylogenetically related to those of Arabidopsis but absent in *P. patens*, suggests that B3-Raf in this clade as a positive regulator of stress signaling had been raised in basal land plants (Figure 1A). Gene expression studies have revealed that the Mp*ARK2* disruption has a greater effect on the ABA-induced gene expression than does the Mp*ARK3* disruption, especially when the gene was co-disrupted with Mp*ARK1* (Figures 5, 7). In the RNA-seq data, there was a significant reduction (−5.13-fold, FDR < 0.05) in the expression of a gene (*Mapoly0072s0050*) encoding ABSCISIC ACID INSENSITIVE5 (ABI5)-like transcription factor in Mp*ark1/2^ge^* but not in Mp*ark1/3^ge^.* Such difference might have caused a drastic reduction in the ABA-induced gene expression in Mp*ark1/2^ge^*.

MpARK1, which forms an independent B3-Raf clade with *P. patens* ARK (Figure 1A), might have functions in the regulation of distinct genes. Unlike Mp*ark2^ge^* and Mp*ark3^ge^*, Mp*ark1^ge^* did not show reductions in *LEA*-like transcript expression but showed reductions in transcripts encoding several bryophyte-specific proteins and proteins for which functions have not been identified such as “desiccation-like protein” (Supplementary Table S2). A distinct function of MpARK1 might be attributed to the domain structures of the polypeptide. The PAS domain found only in the MpARK1/ARK-clade kinases indicates that these B3-Rafs might function as a dimer (Stevenson et al., 2016), whereas other B3-Rafs without PAS domains function as monomers. It has been shown that the PAS domain is important for dimerization of B2-Raf kinases of Arabidopsis (Nguyen et al., 2019), although the functional significance of the dimerization has not been demonstrated. Results of both reporter assays (Figure 1B) and mutant studies (Figures 2-6) indicate that MpARK2 and MpARK3 have a function redundant with that of MpARK1 with respect to the ABA response, indicating that the PAS domain may not be crucial for the ABA response. On the other hand, the EDR1 domain that is common in the B3-Raf kinases of both bryophytes and angiosperms might be important for the function of the B3-Raf molecule. In *P. patens* ARK, a single Ser to Phe substitution in the EDR1 domain was identified in the ABA-insensitive mutant AR7 (Saruhashi et al., 2015), indicating that a small structural change in this domain can affect the function of B3-Raf. The EDR1 domain might be important for interaction with other molecules that regulate the activity of B3-Raf. We recently reported that sensor-histidine kinases (HK) can interact with ARK and that the knockout mutants of HK showed little response to ABA and osmostress in *P. patens* (Toriyama et al., 2022). An N-terminal deletion of ARK to a domain adjacent to the EDR1 domain abolished the interaction with HK, whereas a deletion to the PAS domain alone did not affect interaction with HK or response to ABA (Toriyama et al., 2022). A functional comparison of B3-Raf paralogs of *M. polymorpha* with deletions and point mutations in the EDR1 domain would reveal the role of this domain in interaction with HK and responses to ABA/osmostress.

In this study, we also showed that *M. polymorpha* has B2-Rafs, which are present in angiosperms but not in *P. patens*. Although mutant lines of Mp*B2Raf1/2^ge^* did not exhibit obvious changes in ABA response (Supplementary Figure S3), restoration of ABA-induced gene expression in the *ark* line (Figure 1B) indicates that B2-Rafs might also participate in the ABA response in liverworts. Arabidopsis B2-Rafs appear to be crucial for the ABA response in seeds, but their function might be different from that of B3-Rafs. While B3-Rafs phosphorylate residues in the activation loop of SnRK2 for activation, B2-Rafs phosphorylate a residue near the C-terminus, which can reduce interaction with PP2C-A that negatively regulates SnRK2 (Nguyen et al., 2019). The phosphorylation site for B2-Raf is conserved in SnRK2s of *P. patens* and *M. polymorpha*, although the mode of regulation of SnRK2 in bryophytes might be different from that in Arabidopsis as previously discussed by Komatsu et al. (2013).

### Roles of B3-Raf kinase in gemma dormancy

Dormancy of vegetative organs such as axillary buds and propagules is widely recognized in land plants as a developmental strategy for survival under seasonally changing environmental conditions. The dormancy and its release are controlled by phytohormones such as auxin, cytokinins, abscisic acid, and strigolactones and also by environmental factors such as light, water status, and temperature (Lang, 1987; Horvath et al., 2003). In contrast to the well-defined roles of ABA-regulated factors such as ABI3 (Giraudat et al., 1992), AHG1/3 (Nishimura et al., 2004), and DOG1 (Bentsink et al., 2006) in seed dormancy, the molecular target of ABA for the regulation of dormancy in vegetative tissues has not been clarified. Thus, gemmae formed in gemma cups in *M. polymorpha* can be a useful model for genetic studies of vegetative dormancy, since the developmental processes of the gemmae might be under the control of factors common in embryophytes (Kato et al., 2020). Frequent observation of rhizoid growth in gemmae in gemma cups of Mp*ark1*^ge^ and the Mp*ark1*^ge^-derived double mutants Mp*ark1*/*2^ge^* and Mp*ark1*/3*^ge^* but not in Mp*ark2*^ge^ and Mp*ark3*^ge^ single mutants indicates that Mp*ARK1* plays a specific role in dormancy in gemmae (Figure 3). A higher germination rate observed in Mp*ark1*^ge^ than in Mp*pyl1^ge^* might indicate that the effect of Mp*ARK1* on gemma dormancy includes an ABA-independent process. This notion is consistent with the results of RNA-seq analysis showing a limited correlation between Mp*ARK1*- and Mp*PYL1*-regulated genes (Figure 6A). Furthermore, analysis of ABA-induced gene expression of Mp*ABI3*, which is thought to be a key regulator of ABA-triggered gemma dormancy (Eklund et al., 2018), revealed that, although expression of the Mp*ABI3* transcript was significantly reduced in the Mp*ark1*/*2^ge^* and Mp*ark1*/3*^ge^* double mutants, there was only a slight reduction observed in Mp*ark1*^ge^ (Supplementary Figure S9). These results indicate that the alleviation of gemma dormancy in Mp*ark1*^ge^ was achieved independent of the function of MpABI3. Further analysis of ABA- and B3-Raf-regulated genes should lead to the identification of key factors for the regulation of dormancy in vegetative tissues.

## Data availability statement

The datasets presented in this study can be found in online repositories. The names of the repository/repositories and accession number(s) can be found at: https://www.ddbj.nig.ac.jp/, DRA014012.

## Author contributions

DT and YS: conceptualization. AJ, YY, MI, TG, NY, HK, and KI: investigation. AS: data analysis. AJ, YS, and DT: writing and editing. DT, KI, and YS: funding acquisition. All authors contributed to the article and approved the submitted version.

## Funding

This work was supported by the Program for the Strategic Research Foundation at Private Universities (S1311017) and Grant-in-Aid for Scientific Research (Nos. 20K06680, 18H04774, JP15H05955, and 19H03247) of the Ministry of Education, Culture, Sports, Science, and Technology (MEXT) of Japan.

## Conflict of interest

The authors declare that the research was conducted in the absence of any commercial or financial relationships that could be construed as a potential conflict of interest.

## Publisher’s note

All claims expressed in this article are solely those of the authors and do not necessarily represent those of their affiliated organizations, or those of the publisher, the editors and the reviewers. Any product that may be evaluated in this article, or claim that may be made by its manufacturer, is not guaranteed or endorsed by the publisher.

## Footnotes

^1^ https://phytozome.jgi.doe.gov/pz/portal.html
