# Supplementary material for: Differential regulations of abscisic acid-induced desiccation tolerance and vegetative dormancy by group B3 Raf kinases in liverworts
Source: Front Plant Sci. 2022 Jul 28;13:952820. doi: 10.3389/fpls.2022.952820 (PMC9370073; doi:10.3389/fpls.2022.952820)
Supplement: Supplementary file 1 [file Data_Sheet_1.PDF]

Mp*ARK1*

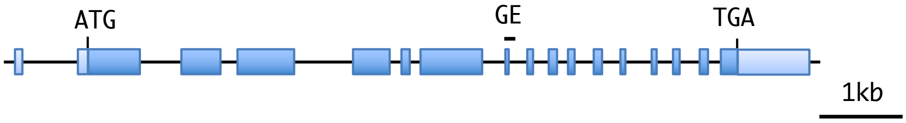

WT GTTCATATGGAAAGGTGTACCGTGCAGATTGGCAGGGAT
V H M E R C T V Q I G R D

Mp*ark1*^{ge} GTTCATATGGAAAGGTGTACCGTGCAAGATTGGCAGGGAT
V H M E R C T V Q D W Q G

Mp*ARK2*

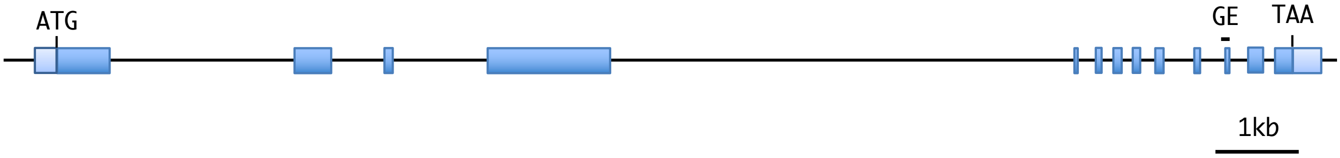

WT GGCAACTCTACGGCAACCCTGGGCAGGGATGAACCCCAT
G N S T A T L G R D E P H

Mp*ark2*^{ge} GGCAACTCTACGGCAACCCTGGGATGTTGGCTTTCAACTGCCAGGGAT
G N S T A T L G C W L S T A R D

Mp*ark1/2*^{ge} a GGCAACTCTACGGCAACCCTGGGGCAGGGATGAACCCCA
G N S T A T L G Q G *

Mp*ark1/2*^{ge} b GGCAACTCTACGGCAACC-----CAGGGATGAACCCCA
G N S T A T Q G *

Mp*ark1/2*^{ge} c GGCAACTCTACGGCAACCCTGG-CAGGGATGAACCCCA
G N S T A T L A G M N P

Mp*ARK3*

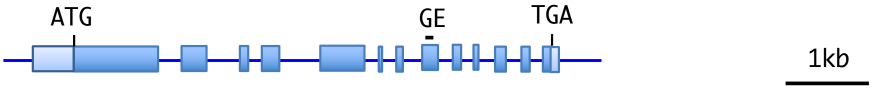

WT CTATTGTCACCGAGTACCTGCCCAGAGGTAGCTTGTTTA
L L S P S T C P E V A C L

Mp*ark3*^{ge} CTATTGTCACCGAGTACCTGCCCCAGAGGTAGCTTGTTTA
L L S P S T C P R G S L F

Mp*ark1/3*^{ge} a,c CTATTGTCACCGAGTACCTGCCCCAGAGGTAGCTTGTTTA
L L S P S T C P R G S L F

Mp*ark1/3*^{ge} b CTATTGTCACCGAGTACCTG-CCAGAGGTAGCTTGTTTA
L L S P S T C Q R *

Figure S1. Gene structures of Mp*ARK1*, Mp*ARK2* and Mp*ARK3* and positions of genome editing in the mutants shown in Figure 2. In each diagram, exons are shown by boxes. Positions of the start codon (ATG), stop codons and the gRNA targets (GE) in the protein kinase domain are marked. The target sequences are underlined in the nucleotide sequences of wild type (WT). Altered nucleotide and amino acids in the mutants are shown in red.

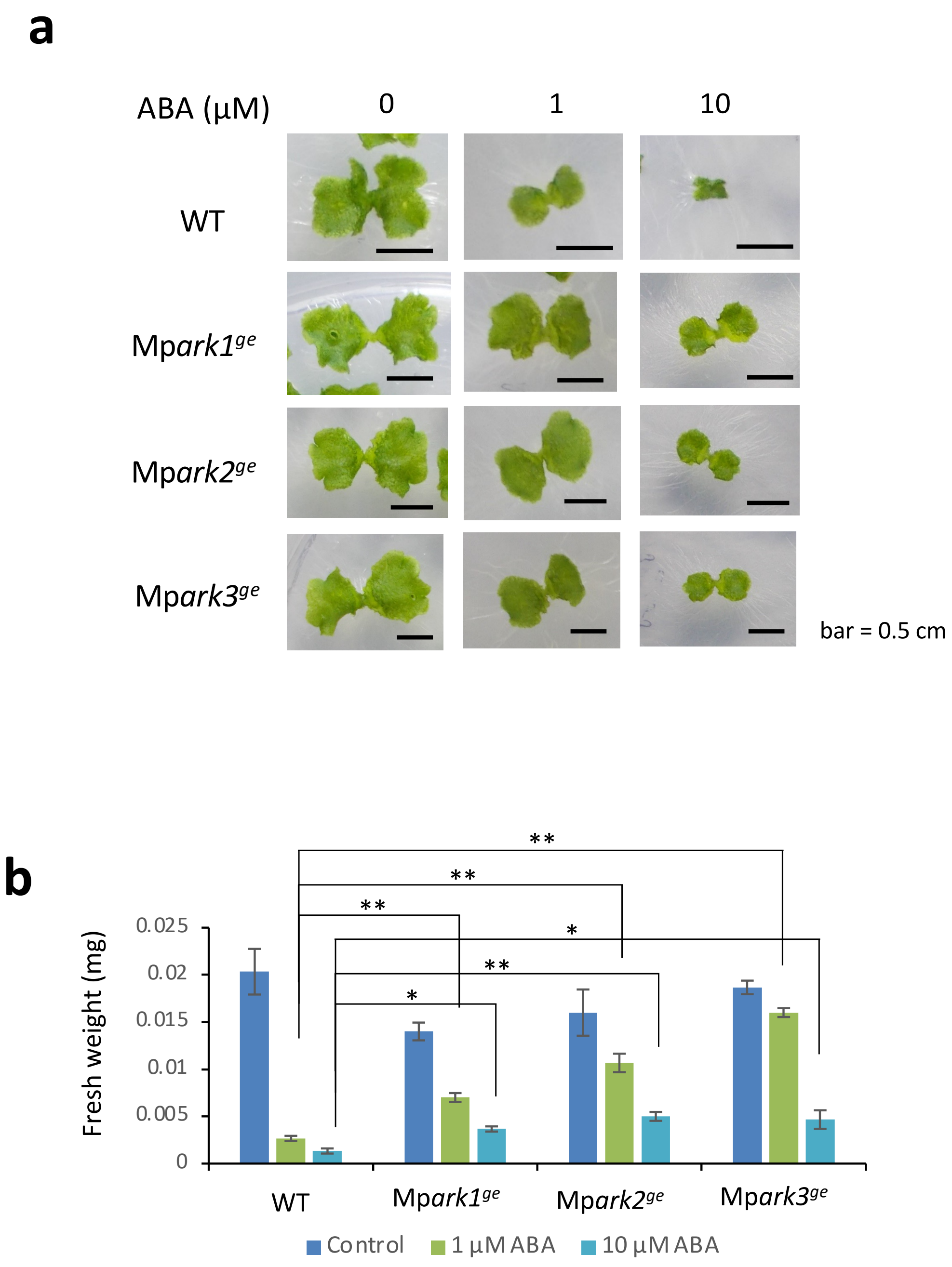

Figure S2. Growth responses to ABA in gemmalings of WT and the single mutant lines of Mp*ARK1*, Mp*ARK2* and Mp*ARK3*. (**a**) Gemmae of WT and the mutant lines were planted on medium without ABA (Control) or that containing 1 or 10 μM ABA and grown for two weeks. (**b**) Fresh weight of the two-week-old gemmalings was plotted on histograms. Error bars indicate +/- SE. *$p<0.05$, ** $p<0.01$ by t-test (n=3).

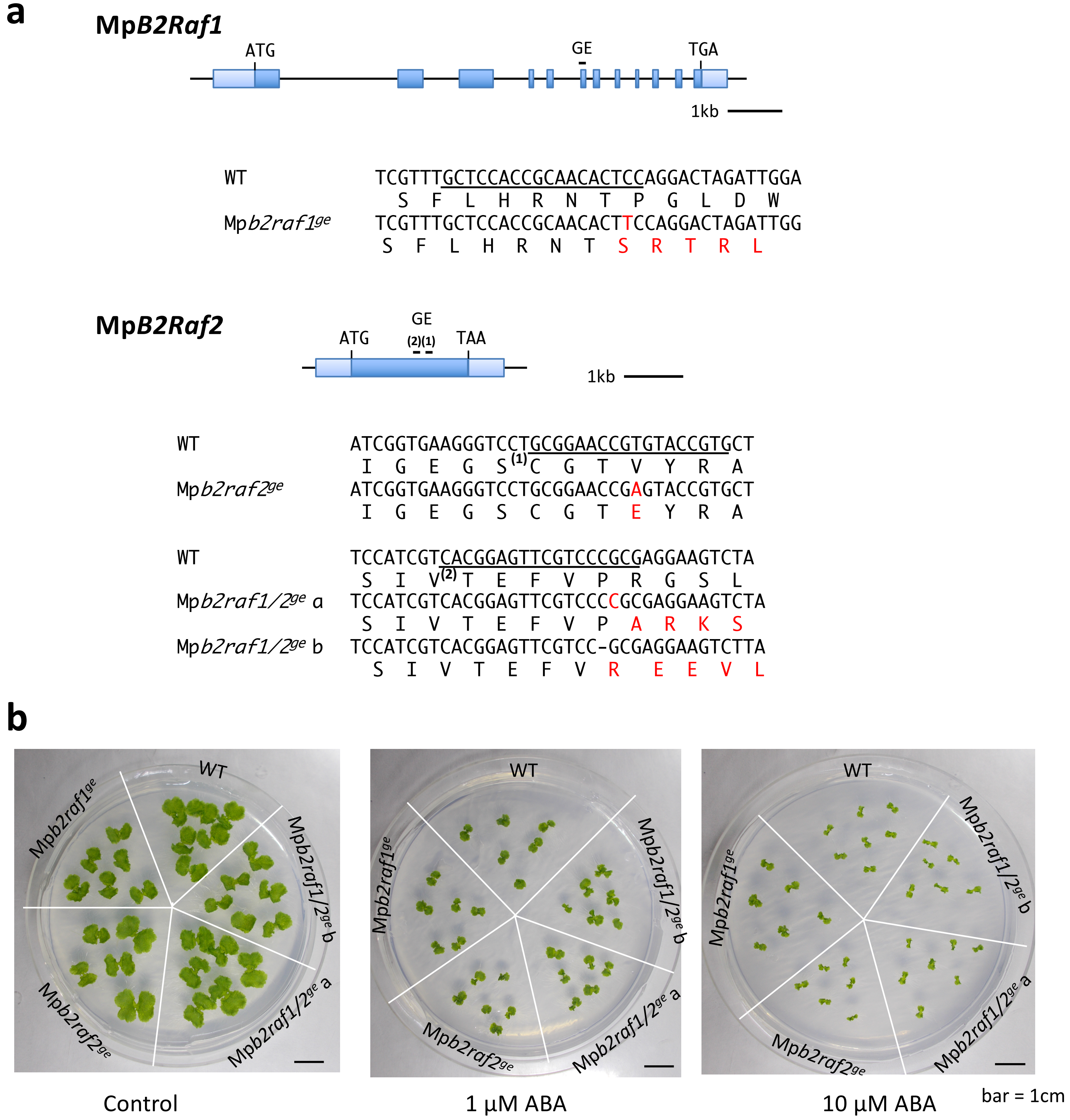

Figure S3. ABA sensitivity of genome-editing lines of group B2 Raf kinases in *M. polymorpha*. **(a)** Gene structures of Mp*B2Raf1* and Mp*B2Raf2* and positions of genome editing in the mutant lines. Exons are shown by boxes in each diagram and the positions of the start codon (ATG), stop codons and the gRNA targets (GE) in the protein kinase domain are marked. The target sequences are underlined in the nucleotide sequences of wild type (WT). Altered nucleotide and amino acids in the mutants are shown in red. First, single mutants of Mp*B2Raf1* (Mp*b2raf1*^{ge}) and Mp*B2Raf2* (Mp*b2raf2*^{ge}) were made, and the Mp*b2raf1*^{ge} line was re-transformed to obtain Mp*b2raf1/2*^{ge}a and Mp*b2raf1/2*^{ge}b lines. **(b)** Growth responses to ABA in gemmalings of WT and the mutant lines. Gemmae of WT, one line each of single disruptants of Mp*B2Raf1* and Mp*B2Raf2*, and two lines of double mutants were planted on medium without ABA (Control) or that containing 1 or 10 µM ABA.

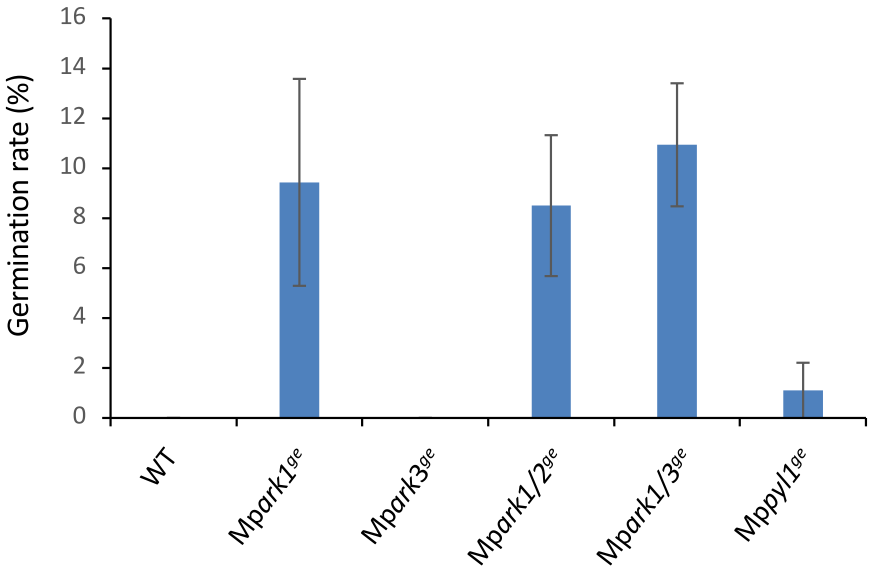

Figure S4. Germination rate of gemmae in gemma cups of WT and genome editing lines. Gemmae isolated from a gemma cup located at the most basal position in the thallus of seventeen-day-old gemmaling were tested. Germination of rhizoids from the gemma was analyzed by staining with propidium iodide. The experiment was carried out on a date different from that shown in Fig. 3. Error bars indicate +/- SE of the mean (n = 3).

a

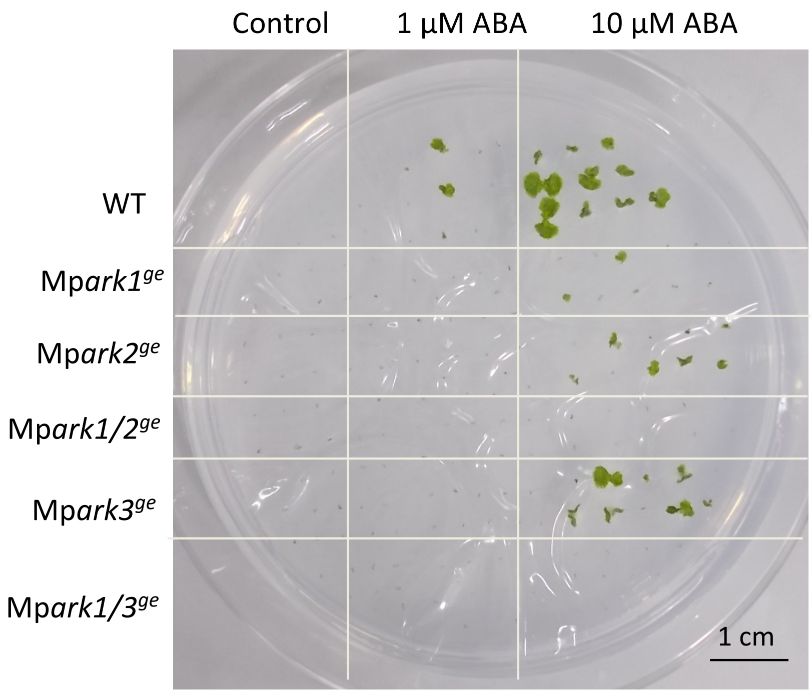

b

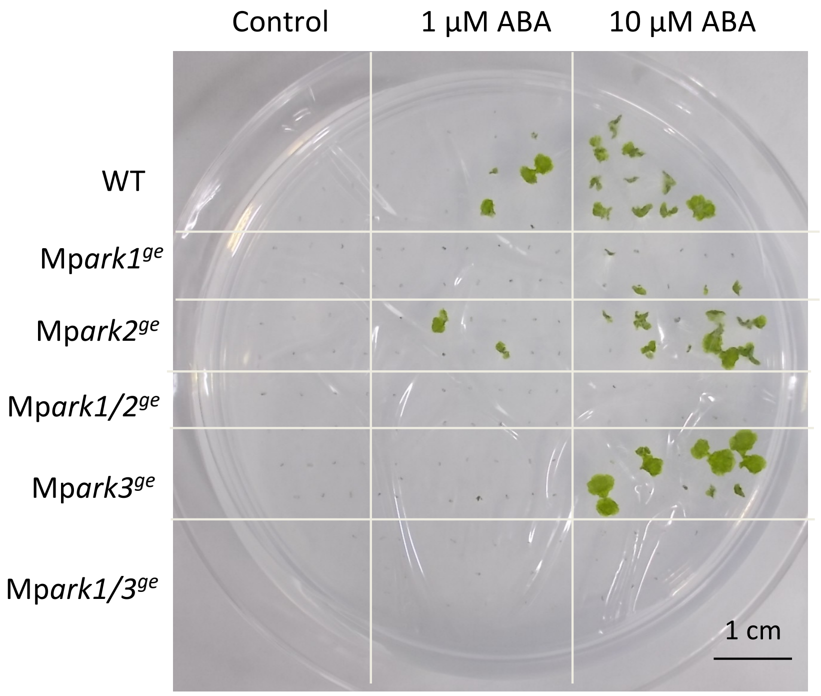

Figure S5. Effect of ABA on desiccation tolerance in *M. polymorpha.* Gemmae cultured for one day with or without 1 or 10 µM ABA were transferred into a container containing silica gel and dried for two days. After rehydration, the gemmae were then transferred onto a fresh agar medium and cultured for 10 days to determine survival. Both **a** and **b** are repetition of experiments shown in Fig. 4.

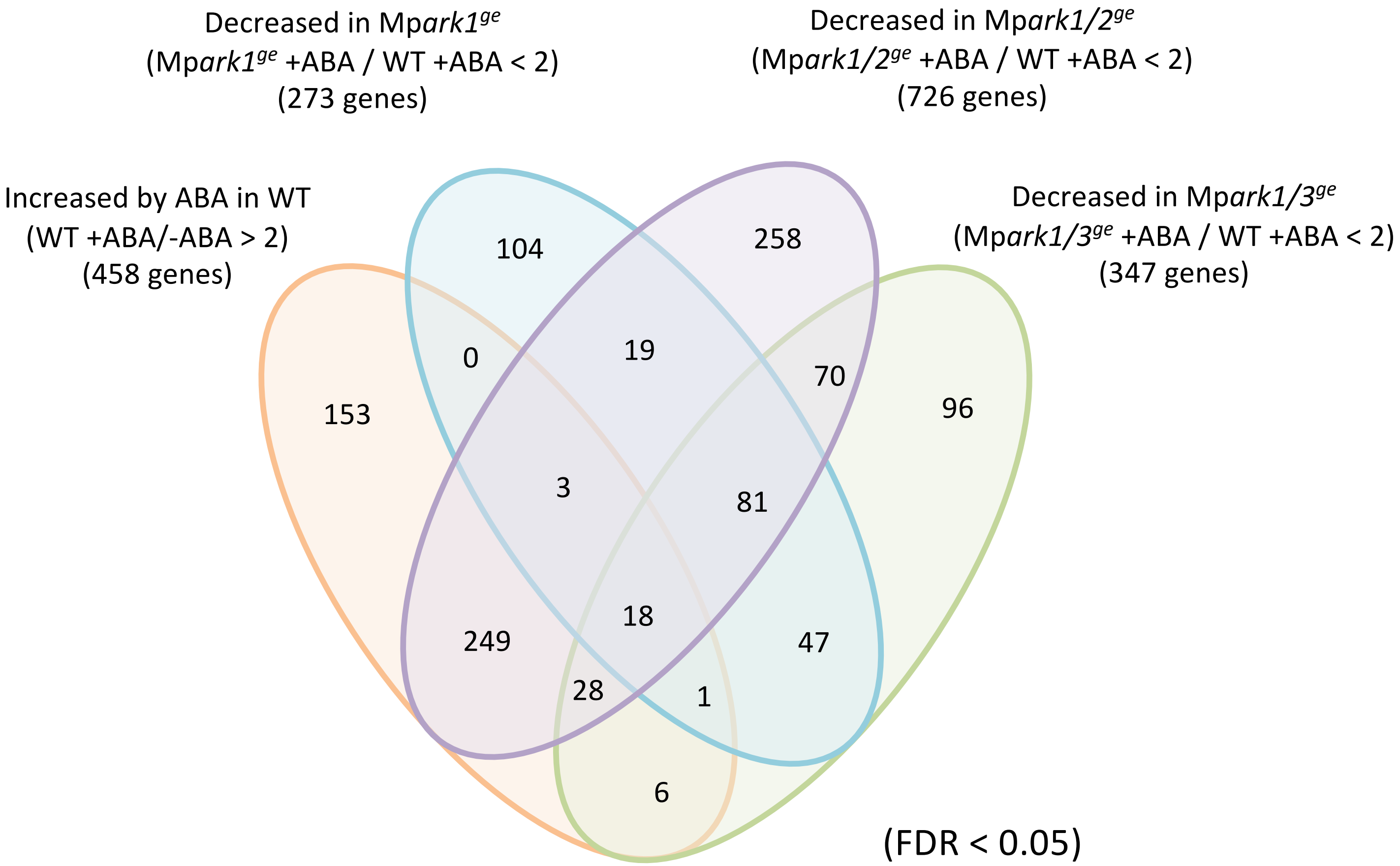

Figure S6. Venn diagram showing the relationship between the ABA-induced genes and the B3-Raf-regulated genes.

a

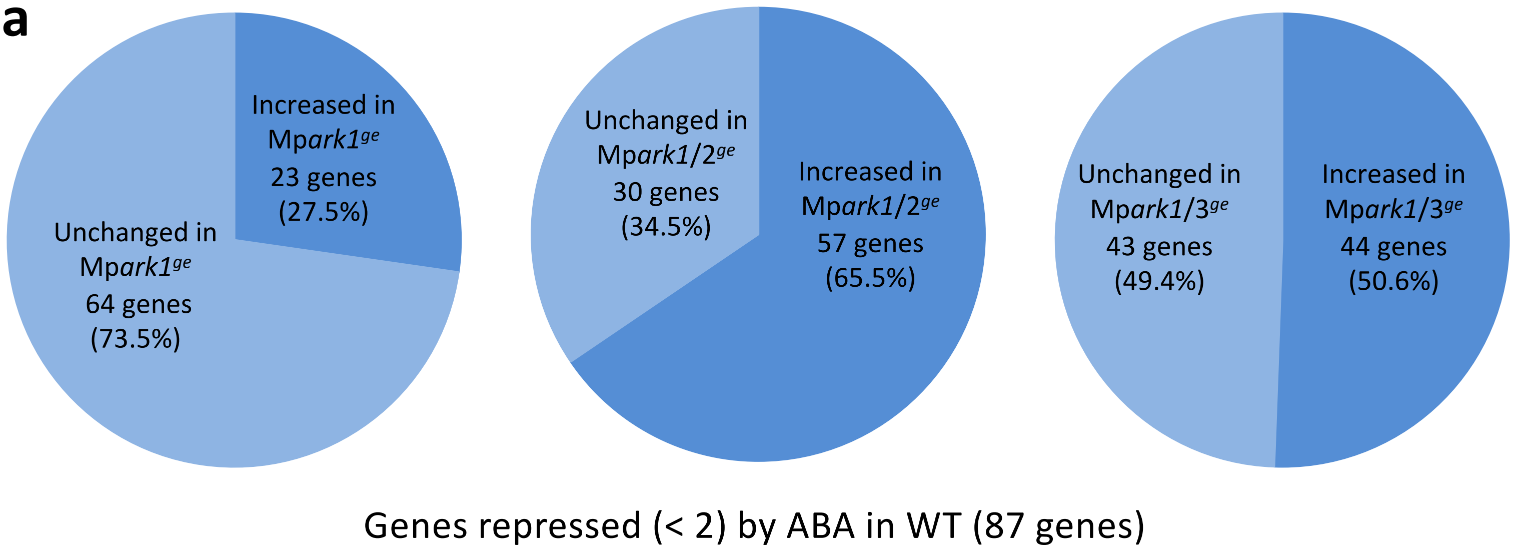

b

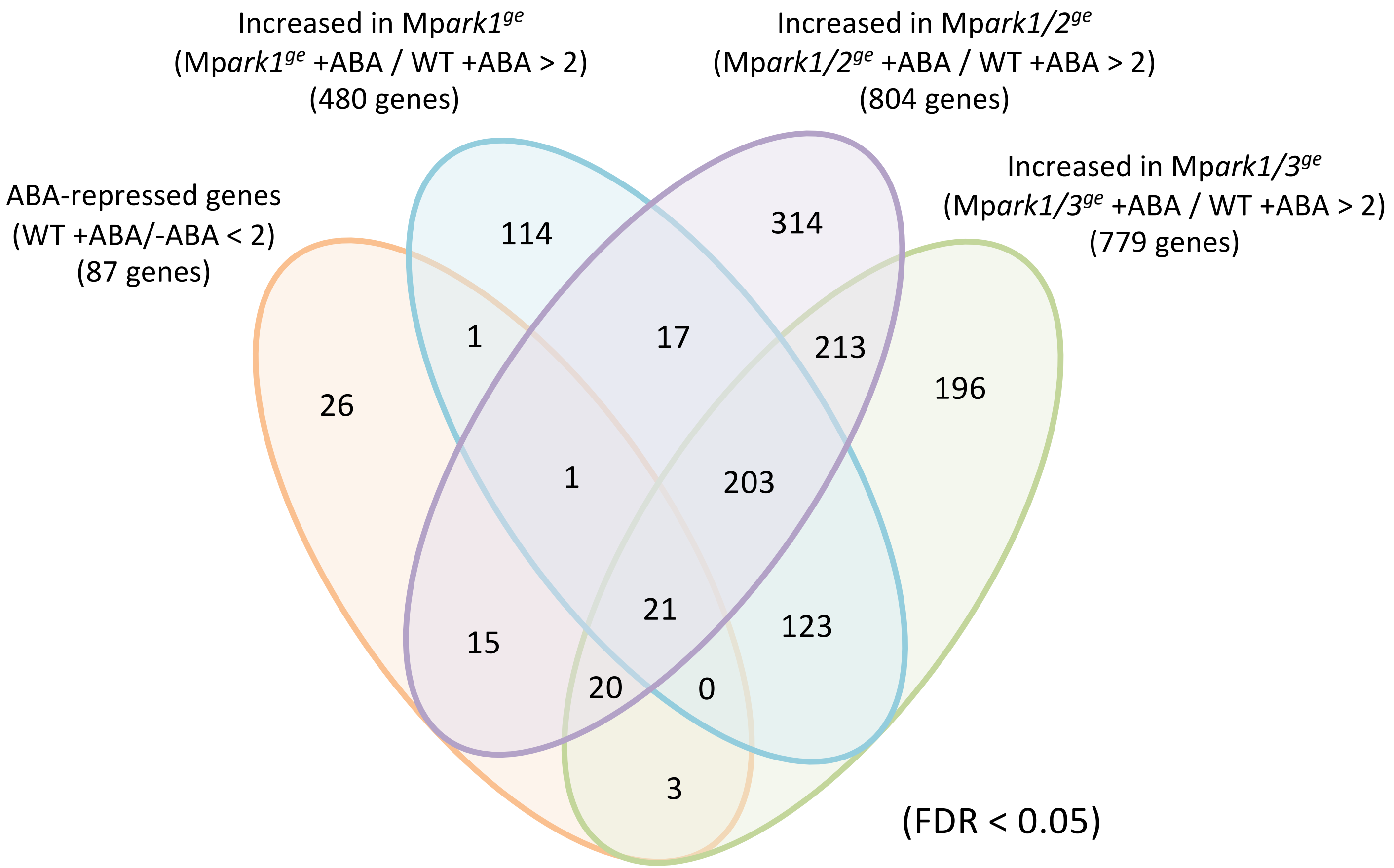

Figure S7. Effect of B3-Raf disruptions on ABA-repressed gene expression. **(a)** Percentage among the 87 ABA-repressed genes (< 2) for which expression was increased (> 2) in each genome-editing line (FDR < 0.05). **(b)** Venn diagram showing the relationship between the ABA-repressed genes and the B3-Raf-regulated genes.

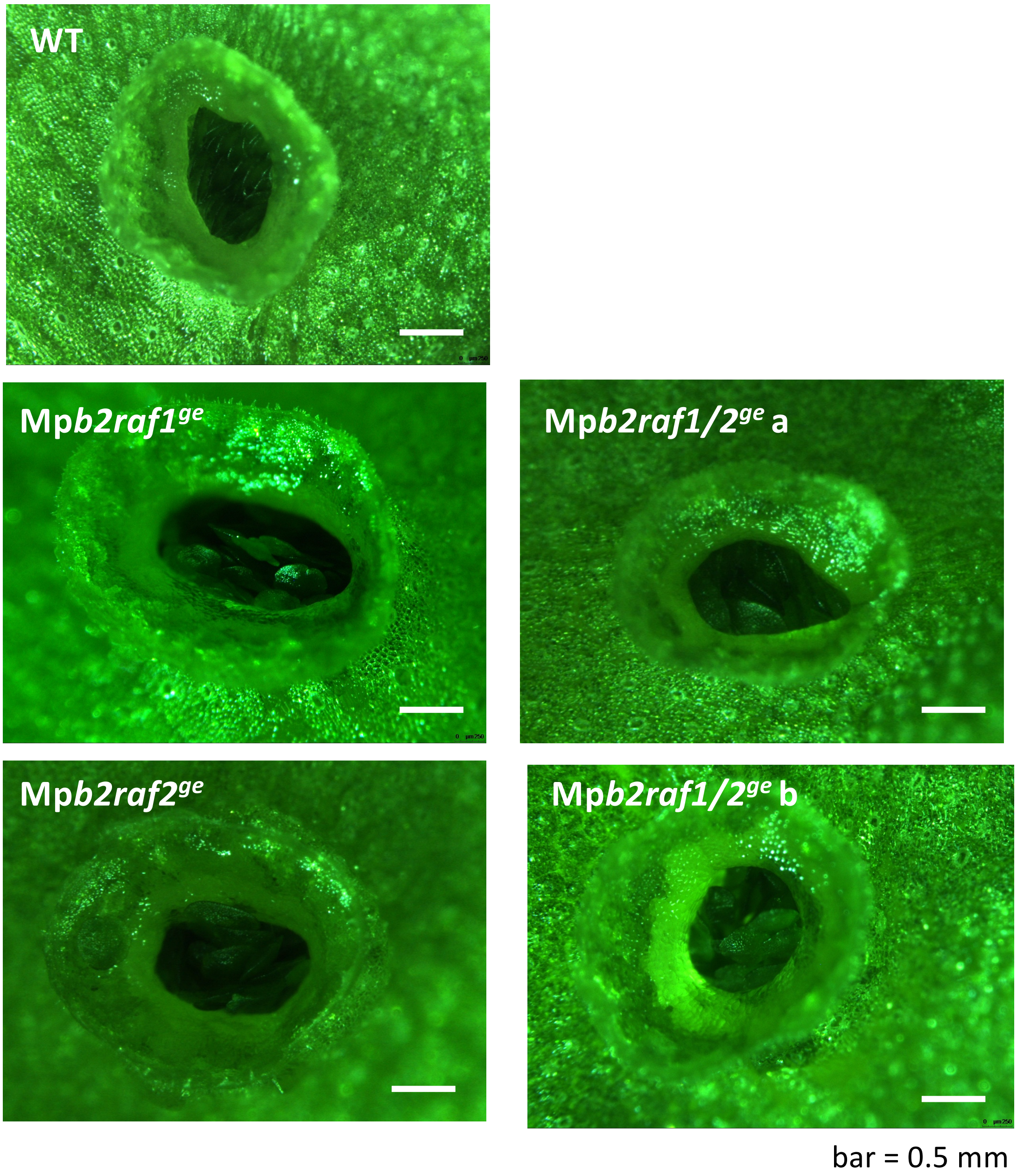

Figure S8. Appearance of gemmae in gemma cups of wild type (WT) and genome-editing lines of group B2 Raf kinases. Details of genome editing are shown in Fig. S3.

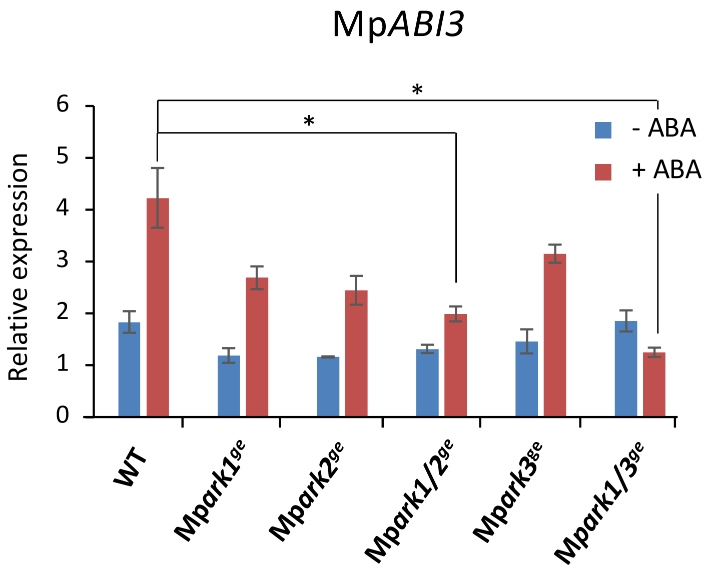

Figure S9. ABA-induced expression of Mp*ABI3* transcripts in wild type and genome editing lines of *Marchantia polymorpha* determined by SYBR green-based quantitative PCR analysis. Error bars indicate SE of triplicates. *$p < 0.05$ in the t-test (n = 3) compared with the values of WT.
