# Supplementary material for: Differential regulations of abscisic acid-induced desiccation tolerance and vegetative dormancy by group B3 Raf kinases in liverworts
Source: Front Plant Sci. 2022 Jul 28;13:952820. doi: 10.3389/fpls.2022.952820 (PMC9370073; doi:10.3389/fpls.2022.952820)
Supplement: Supplementary file 2 [file Table_1.DOCX]

| **Table S1.** Oligonucleotide primers used for quantitative RT-PCR. | | | |
| --- | --- | --- | --- |
| gene names | gene ID | forward (5' to 3') | reverse (5' to 3') |
| Mp*EF1* | *Mapoly0024s0116*  (*Mp3g23400*) | AAGCCGTCGAAAAGAAGGAG | TTCAGGATCGTCCGTTATCC |
| Mp*LEAL1* | *Mapoly0112s0030*  (*Mp4g09300*) | GCCAAAGACAAGGCTGTAGA | CAATGGTGGATTGGTCCTACT |
| Mp*LEAL3* | *Mapoly0035s0082*  (*Mp6g02960*) | GAGCAGACCAAGAATTTTGGAGC | CAGCAGCCTTGTCCTTGGTC |
| Mp*LEAL5* | *Mapoly0087s0015*  (*Mp4g05760*) | CTGTGCAAGGCAAGGATTACG | GAGGTGTATTGACCGGCAGAAC |
| Mp*LEAL6* | *Mapoly0027s0114*  (*Mp5g05120*) | ACGAGTGAAGGAGGTGGTTCTAAG | CACCTGATCCTTGGCACTCTGG |
| *MpABI3* | *Mapoly0086s0035*  (*Mp5g08310*) | GTGACGTTCCAGCGTACTCGAG | CATGTCCATAGCGTGGTGGTCC |
